# A survey on the incidence of common musculoskeletal side effects among the patients taking long-term anti-ulcerant therapies in Bangladesh

**DOI:** 10.1016/j.toxrep.2022.09.007

**Published:** 2022-09-17

**Authors:** Shuvo Mitra, Md. Saddam Hussain, Rubiya Rahman, Md. Abdus Salam, Tanoy Mazumder, Sarmin Farzana

**Affiliations:** a Department of Pharmacy, Faculty of Science, Noakhali Science and Technology University, Noakhali 3814, Bangladesh; b Pharmacy Discipline, Khulna University, Sher-E-Bangla Road, Khulna 9208, Bangladesh; c Department of Radiology and Imaging, Bangabandhu Sheikh Mujib Medical University, Dhaka 1000, Bangladesh

**Keywords:** PPIs, Proton pump inhibitors, H2-blockers, Histamine type 2 receptor blockers, BMD, Bone mineral density, GIT, Gastrointestinal tract, HCL, Hydrochloric acid, NDMA, N-nitrosodimethylamine, DNA, Deoxyribonucleic acid, IC HCY, Intracellular homocysteine, EC HCY, Extracellular homocysteine, Ca, Calcium, Mg, Magnesium, GERD, Gastroesophageal reflux disease, ZES, Zollinger-Ellison syndrome, NSAID, Non-steroidal anti-inflammatory drug, HP, Helicobacter pylori, PU, Peptic ulcer, BMC, Bone mineral content, IBM, International business machines, GDP, Gross domestic product, ALP, Alkaline phosphatase, OCN, Osteocalcin, PTH, Parathyroid hormone, BMI, Body mass index, ERCP, Endoscopic retrograde cholangiopancreatography, Proton pump inhibitors (PPIs), H2 blockers, BMD, Fracture, Bone pain

## Abstract

**Background:**

Proton pump inhibitors (PPIs) and H_2_ blockers are commonly prescribed medications to treat ulcers in the stomach and the upper part of the small intestine and prescribed for some other common gastrointestinal complications such as gastroesophageal reflux disease, esophagitis, irritable bowel syndrome, and dyspepsia. Previous studies claimed that, apart from other side effects, these anti-ulcerant therapies significantly altered bone mineral density by interfering with intestinal reabsorption of minerals and vitamin B12, and the most widely prescribed PPIs were significantly associated with increased risks of hip and spine fractures. However, the potential skeletal side effects of these antiulcerants are unknown in Bangladesh.

**Methods:**

To examine safety concerns of anti-ulcer therapies and their impact on musculoskeletal health among patients in Bangladesh, the present work surveyed 200 patients in five different hospitals from December 2019 to February 2020.

**Results:**

The current study revealed that most respondents (95 %) received PPIs for gastrointestinal indications while the rest were taking H2 receptor antagonists for their gastric ailments. Most patients taking PPIs alone (> 3 years; 95 % of respondents) claimed some unusual musculoskeletal side effects, such as weakness, flank pain, spasm of hands and feet, muscle aches, numbness, and tremor. About 61 % of patients taking PPIs experienced low back pain whereas the respondents with neck pain and knee joint pain were 10 % and 7 %, respectively. However, few osteopenia and osteoporotic incidences have been also recorded. Although further studies are required to confirm the impact of these antiulcerants on the bone, these patient responses suggest that these musculoskeletal side effects might have some links with altered bone metabolism.

**Conclusions:**

It is possible that anti-ulcerant therapies may worsen the bone metabolism of patients suffering from osteoporosis or other bone disorders, and awareness and precautions should be raised among the patients and clinicians for the careful administration of PPIs to patients suffering from bone disorders.

## Highlights

• Anti-ulcerant therapies can interfere with intestinal reabsorption of minerals and vitamin B12.
• PPIs are associated with increased risks of hip and spine fractures.
• Patients taking PPIs (>3 years; 95% of respondents) claimed unusual musculoskeletal side effects.
• Anti-ulcerant therapies may worsen the bone metabolism of patients suffering from osteoporosis or other bone disorders.
• Awareness should be raised among the patients and clinicians for the careful administration of anti-ulcerant therapies.

## Graphical Abstract

**Figure:**
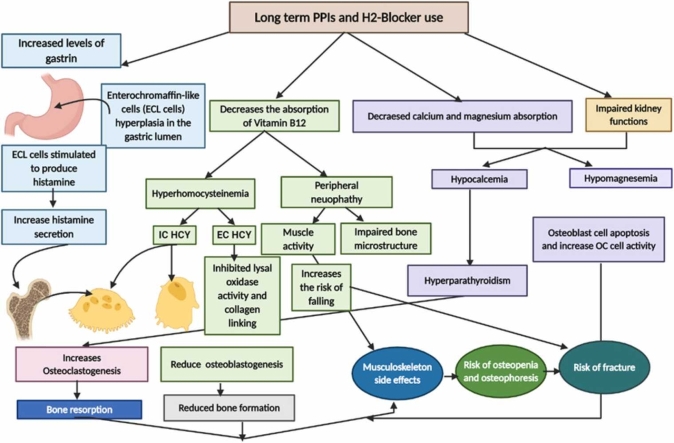

## 1 Introduction

The gastric secretions are necessary for food digestion and optimal absorption of vitamins and minerals which are important for the functions of the different tissues/organs including the musculoskeletal system and its functioning to support the body weight and posture [1]. Highly concentrated hydrochloric acid (HCL) constitutes the major volume of the gastrointestinal tract (GIT) secretion, which serves two major functions: killing the ingested microorganisms through food and facilitating the digestion and metabolism of the foodstuffs (starch, lipid and protein) by activating the enzymes secreted from the liver [1]. However improper release and actions of gastric secretions manifest in different pathological conditions such as dyspepsia, gastroesophageal reflux disease (GERD), and Zollinger Ellison syndrome (ZES), which, untreated with the proper medications, can later initiate ulceration in the stomach and upper intestine [2]. Meanwhile, a thick layer of mucus protects the tissue of the stomach from the deleterious and corrosive effects of gastric secretion. However, if the mucus layer is worn away and stops functioning efficaciously, the secreted gastric acid can cause a disturbance in the stomach and upper intestine tissue and may lead to ulceration later on [2].

The currently available anti-ulcerant or acid-suppressive drugs have been found effective for healing the ulcers in the stomach and the upper part of the small intestine and while also effective against other indications including *Helicobacter pylori* infection, dyspepsia, GERD, and ZES [3]. Though non-systemic antacids (aluminum or magnesium hydroxide and calcium carbonate) have been effectively prescribed and delivered with or without mucosal protective agents to have some instant and local relief from the ulceration. However, for the utmost relief, the selective histamine type 2 receptor blockers (H2-blockers) and the proton pump inhibitors (PPIs) are the two most suitable classes/groups for the effective treatment of ulceration in the stomach and the upper small intestine [3].

Among these two groups of medications, proton pump inhibitors are the most widely prescribed for the treatment of gastrointestinal disorders ranging from mild irritations to peptic ulcers (PU) because of their superior acid-suppressive properties and excellent safety index than prior antiulcerants [4], [5]. H2-blockers have some safety concerns [6], [7], [8], recent reports say the presence of unacceptable levels of N-nitrosodimethylamine (NDMA) [9], [10], which is a hepatotoxic and carcinogenic agent with DNA mutation properties [9], [10]. Considering all these concerns, PPIs have become the first consideration for treating gastrointestinal disorders. As a result, the inexpensive generic formulations of PPIs have already taken the maximum share of the antiulcerant prescriptions [4]. On the other hand, this cost reduction has also adversely impacted the quality use of PPIs, including patient’s dependency on PPIs and physicians’ tendency to overprescribe PPIs or prescribe the PPIs at an unrevised dose over time even if there are no or mild symptoms of GIT problems [11]. These issues can ultimately increase the patient's risk of experiencing adverse events in different body systems resulting from the long-term use of PPIs [12], [13], [14], [15], [16], [17], [18].

Previous studies have presented evidence that long-term PPI administrations to patients can lead to hypomagnesemia, hypocalcemia, hypoparathyroidism, impaired kidney and gut microbe functions and vitamin B12 deficiencies [12], [17], [18], all of which can increase the patient's risk of experiencing musculoskeletal side effects and in the long run, may generate the risk of experiencing osteoporosis and fractures in different bone sites [18]. Interestingly, previous work has shown that while long-term use of PPI medications is significantly associated with an increased risk of hip fracture, any-site fracture and spine fracture, no evidence of fracture has been noticed in patients with H2 receptor antagonist exposure [19], [20]. However, despite these reports, the potential skeletal side effects of these antiulcerants are unknown for Bangladesh patients.

The present study, by conducting a structured questionnaire survey, aimed to assess the prevalence of present consumption, probable complications or adverse reactions and the impact on musculoskeletal systems of PPI usage in Bangladesh. This study also evaluated some factors such as the family history of fracture incidence, some physical conditions as well as risk factors, diseases and medications that may have undesirable repercussions on bone health.

## 2 Methods

### 2.1 Study design and sampling

This survey was carried out among Bangladesh patients taking two common anti-ulcerant therapies for a long term (at least three years) so to investigate the present consumption, health-hazardous patterns and their potential musculoskeletal side effects. Depending on the easy accessibility and easy availability of participants, the respondents were chosen from five different hospitals in Chattogram, Bangladesh (BGC Trust Medical College; Patiya Central Hospital; Patiya Health Complex; Chevron Diagnostic and Health Centre; and Neuron Diagnostic and Health Centre Metro Lab and Gastro Hospital) between December 2019 and February 2020. A simple random sampling technique was used for the selection of study participants. Besides, to ensure the respondent's least knowledge about the medications and their effects, this current study only considered respondents who have completed at least their higher secondary education (HSC). A total of 200 participants were selected, including 130 male participants and 70 female participants. After having their consent with the proper explanation of the research objectives and assurance of the confidentiality of their participation, the pre-designed questionnaires were explained to participants. The study protocol was approved by Noakhali Science and Technology University, Bangladesh.

### 2.2 Data collection

A face-to-face interview was conducted by the field researchers. During the interview in person, the interviewers translated the questionnaire from English to Bengali to make the questions easily understandable to the respondents. Interviewers clarified any doubts that participants had and explained the symptoms and medical terms in their familiar names and terms. The interview lasted 45–60 min and included a range of questions about age, education, income and health status of respondents, history of diagnosis and treatment protocols for gastrointestinal disease, other drug information and complications (vitamin and mineral deficiencies; musculoskeletal and other side effects) they experienced during the therapy.

### 2.3 Data analysis

All data obtained were coded into a Microsoft Excel spreadsheet and categorized into groups according to their background. The data were summarized as counts (or percentages) and represented as percentage ± SEM (standard error of the mean), where possible descriptive statistics (p-value) were computed by using SPSS version 26 (IBM, Armonk, NY, USA). P < 0.05 was considered statistically significant.

## 3 Results

### 3.1 Demographic and socioeconomic status

The current survey was conducted among individuals who had been receiving frequent antiulcerants at least for the last 3 years from the date they were surveyed. As Table 1 shows, compared to other groups, the majority of respondents taking antiulcerants were from the middle-class group (economic class are defined based on the gross national GDP and economic circumstances of individuals). While considering the age group of the respondents, results showed that most of the respondents were more than 40 years old, whereas a small fraction (5 %) of respondents were between the 30–40 age group (Table 1). The respondents included both sexes, where most respondents were from the male group (62 %), and the rest were female in gender. Table 1 shows that most respondents had completed at least their HSC education, while 44 % of respondents were fallen between HSC and the graduation level (Table 1). The professional status of the respondents was also considered during this survey, where the results showed that almost one-third of the respondents had no jobs or were retired, while most respondents were job holders. Finally, as people's religious belief also affects their food habit, physical activities, and disease prognosis and treatment, this survey also considered this status where Muslim respondents recorded the highest value (84%) compared to other religions (Table 1).

**Table 1:** Demographic and socioeconomic status of the respondents.

Parameters	Subgroups	Percentage ()%	SEM (between sub-groups)	P-value (between sub-groups)
**Economic status**	Upper class	0	18.48	0.269 ^NS^
	Upper middle	10		
	Middle	80		
	Lower middle	10		
**Age**	30–40 years	9	41.00	0.437 ^NS^
	More than 40 years	91		
**Gender**	Male	62	12.00	0.150 ^NS^
	Female	38		
**Education Level**	Up to HSC	56	6.00	0.076 ^NS^
	HSC to Graduation level	44		
**Occupation**	Non-job Holder	27	16.19	0.176 ^NS^
	Job Holder	64		
	Retired	9		
**Religion**	Muslim	83	19.51	0.290 ^NS^
	Hinduism	13		
	Buddhism	3		
	Christian	1		

Values are represented by percentages and SEM is calculated for the subgroups, and the total number of respondents was 200. NS stands for statistically not significant at a p-value > 0.05 for all parameters. Economic class is defined based on the gross national GDP and economic circumstances of individuals.

### 3.2 Physical and habitual status

Table 2 demonstrated that most respondents were of normal weight while the overweight and underweight respondents were approximately 30 % and 2 % respectively (based on the BMI calculation for the respondents). About 66 % of respondents never smoked a cigarette (non-smokers), 29 % were regular smokers (fewer than 20 cigarettes a day) and the rest of the respondents were chain smokers (more than 20 cigarettes a day). Moreover, the pattern of alcohol intake was considered among the respondents, and the results indicated that 95 % of respondents were found to be non-alcoholic, with only 2 % of respondents being severely alcoholic. Considering the dietary habits of the respondents, the findings revealed that carbohydrate-rich foods (rice, bread and vegetables), protein-rich foods (meat, fish, vegetables) and lipid-rich foods (animals and vegetables) imbibed were 75.5 %, 10 %, and 14.5 % respectively of their daily meals. The results of the study also showed that almost all the respondents were not concerned about physical exercise apart from just walking and running, but 4.5 % replied they had undergone some structured physical exercise.

**Table 2:** Physical and habitual status of the respondents.

Parameters	Subgroup	Percentage (%)	SEM	P-value (between the sub-groups)
**Health status (based on BMI)**	Underweight	2	19.13	0.223 ^NS^
	Normal Bodyweight	68		
	Overweight or obese	30		
**Cigarette smoking status**	Non-Smoker	66	17.74	0.201 ^NS^
	Normal Smoker (< 20 cigarettes/day)	29		
	Chain smoker (> 20 cigarettes/day)	5		
**Alcohol**	Non-alcoholic	95	30.84	0.393 ^NS^
	Regular alcoholic	3		
	Severe alcoholic	2		
**Food habit**	Carbohydrate-rich	75.5	21.09	0.255 ^NS^
	Protein-rich	10		
	Fat-rich	14.5		
**Exercise**	No exercise	95.5	45.50	0.470 ^NS^
	Non-traditional	4.5		

Values are represented by percentages and SEM is calculated for the subgroups, and the total number of respondents was 200. NS stands for statistically not significant at a p-value > 0.05 for all parameters. Respondents with 18.5; 18.5—24.9; 25.0—29.9; or 30.0 and above BMI values were labeled with underweight, normal, overweight or obese, respectively.

### 3.3 Gastrointestinal (GI) disorders in respondents and techniques applied for the diagnosis

Table 3 shows a clear projection of respondents taking antiulcerant treatments for different GI ailments, with the majority being diagnosed with gastrointestinal reflux disease (GERD), while 22.5 % of respondents having dyspepsia, 10 % of respondents having esophagitis and a very few numbers of respondents being diagnosed with irritable bowel syndrome (IBS) and peptic ulcer (PU).

**Table 3:** Gastrointestinal problems diagnosed among patients taking PPIs.

Gastrointestinal problems	Number of cases (percentage)	Case per gender (male/female) (percentage)	P-value (between genders)	SEM (between groups)	P-value (between groups)
**Esophagitis**	20 (10.00)	13/7 (65.00/35.00)	0.186 ^NS^	10.98	0.143 ^NS^
**Dyspepsia**	45 (22.50)	26/19 (58.78/42.22)	0.189 ^NS^		
**Gastroesophageal reflux disease (GERD)**	123 (61.50)	76/47 (61.79/38.21)	0.147 ^NS^		
**Irritable bowel syndrome (IBS)**	9 (4.50)	7/2 (77.78/22.22)	0.266 ^NS^		
**Peptic ulcer (PU)**	3 (1.50)	2/1 (66.67/33.33)	0.205 ^NS^		

Values are represented by a percentage and SEM calculated for the groups; the total number of respondents was 200; NS stands for statistically not significant at p-value > 0.05 for all parameters.

The methods of diagnosis for different gastrointestinal (GI) problems in respondents are shown in Table 4. Approximately 85.5 % of the respondents were diagnosed based on physical examination (physicians have confirmed the cases based on the symptoms and history), whereas the 14.5 % prevalence of gastrointestinal disorders was confirmed through the hospital and clinical lab diagnosis.

**Table 4:** Methods of diagnosis of gastrointestinal diseases for the respondents.

Name of the Method	Number of cases (percentage)	Case per gender (male/female) (Percentage)	P-value (between the gender)	SEM (between the group)	P-value (between the group)
**Physical examination**	171 (85.50)	104/67 (60.82/39.18)	0.136 ^NS^	35.50	0.393^NS^
**Lab diagnosis**	29 (14.50)	20/9 (68.97/31.03)	0.231 ^NS^		

Values are represented by a percentage and SEM calculated for the groups; the total number of respondents was 200; NS stands for statistically not significant at p-value > 0.05 for all parameters.

Table 5 represents the list of diagnosis techniques employed for the diagnosis of 29 cases of GI problems. Based on the responses (n = 29 respondents), endoscopic retrograde cholangiopancreatography (ERCP) was most used, while other techniques used were in the following order: gastroscopy > endoscopic ultrasound and pH monitoring > urea breath test.

**Table 5:** Diagnostic techniques employed for lab diagnosis for the respondents (number of respondents = 29).

Name of the technique	Number of cases (percentage)	Cases per gender (male/female) (percentage)	P-value (between genders)	SEM (between groups)	P-value (between groups)
**Gastroscopy**	16 (55.17)	12/4 (75.00/25.00)	0.295 ^NS^	15.70	0.048*
**Endoscopic retrograde cholangiopancreatography (ERCP)**	29 (100)	20/9 (68.97/31.03)	0.231 ^NS^		
**Endoscopic ultrasound**	7 (27.59)	5/2 (71.43/28.57)	0.258 ^NS^		
**P^H Monitoring^**	7 (27.59)	5/2 (71.43/28.57)	0.258 ^NS^		
**Urea breath test**	3 (10.35)	2/1 (66.67/33.33)	0.205 ^NS^		

Values are represented by a percentage and SEM calculated for the groups; the total number of respondents was 29; *p < 0.05, NS stands for statistically not significant at p-value > 0.05.

### 3.4 Types of antiulcerant treatments among the respondents

As shown in Table 6, in Bangladesh, doctors have been prescribing either proton pump inhibitors (PPIs) or H2-blockers for GI ailments. However, 95 % of respondents received PPI-based treatments, while only 5 % of the respondents took H2-blocker-based treatments (ranitidine only).

**Table 6:** The types of antiulcerant treatments used by the study subjects.

Antiulcerant group	Number of cases (percentage)	Cases per gender (male/female) (Percentage)	P-value (between genders)	SEM (between groups)	P-value (between groups)
**H2-blockers**	10 (5.00)	7/3 (70.00/30.00)	0.242 ^NS^	45.00	0.467^NS^
**Proton pump inhibitors**	190 (95.00)	117/73 (61.58/38.42)	0.145 ^NS^		

Values are represented by cases (percentages) and SEM is calculated for the groups. The total number of respondents was 200. NS stands for statistically not significant at a p-value > 0.05 for all parameters.

It is evident from the survey that, among all PPI users (190), most respondents (67 %) received omeprazole, with the second major response being recorded for esomeprazole, and very few respondents were taking rabeprazole and pantoprazole-based treatments (Table 7). This study has also recorded the respondent’s PPI treatment dosage frequencies and treatment patterns. From the responses, both frequently prescribed PPIs, omeprazole and esomeprazole, have been used in patients for more than 5 years, and daily administrations of single and double doses of omeprazole have almost taken the same proportion (Table 7). However, single-dose (per day) esomeprazole therapy has been used more the double-dose treatment. Meanwhile, only 10 respondents received H2-Blockers for GI problems and almost all of them belong to double dose (per day) and short-term administration (Table 7).

**Table 7:** Administration and treatment patterns of H2-blockers and proton pump inhibitors (PPIs) in study subjects.

Name of the antiulcerant	Number of cases (percentage)	SEM	Dosage Frequency (number of dose/day)	Number of cases (percentage)	SEM	Treatment pattern	Number of cases (percentage)	SEM
**H2-blocker (n = 10)**								
Ranitidine (150 mg, oral)	10 (100)	–	Single	2 (20.00)	30.00	Long term	1 (10.00)	40.00
			Double	8 (80.00)		Short term	9 (90.00)	
**Proton Pump Inhibitors (n = 190)**								
**Omeprazole**(20 mg, oral)	128 (67.37)	21.23	Single	75 (58.59)	8.59	Long Term	122 (95.31)	45.31
			Double	53 (41.41)		Short term	6 (4.69)	
**Esomeprazole**(20 mg, oral)	48 (25.26)		Single	31 (64.58)	14.58	Long Term	40 (83.33)	33.33
			Double	17 (35.42)		Short term	8 (16.67)	
**Rabeprazole**(20 mg, oral)	8 (4.21)		Single	5 (62.50)	12.50	Long Term	6 (75.00)	25.00
			Double	3 (37.50)		Short term	2 (25.00)	
**Pantoprazole**(20 mg, oral)	6 (3.16)		Single	3 (50.00)	0.00	Long Term	4 (66.67)	16.67
			Double	3 (50.00)		Short term	2 (33.33)	

Values are represented by cases (percentages) and SEM is calculated for the groups; the total number of respondents was 200; short-term usage (> 3 to < 5 years), long-term usage (> 5 years).

### 3.5 Some common side effects of antiulcerants therapies (PPIs and H2-blockers)

Table 8 represents the common side effects of antiulcerant treatments. Ranitidine users had some common GI discomfort: constipation (50 %), flatulence (10 %) and abdominal discomfort (80 %), while a few of them complained they had insomnia (40 %) and dizziness (30 %)-like side effects once they had started taking ranitidine treatment. Among the PPIs users, two common problems have been recorded for all generic administration of PPIs: headache and nausea (Table 8). Meanwhile, the pantoprazole, omeprazole and rabeprazole users also had dizziness-like side effects (Table 8). With this problem, 62.5 % of rabeprazole users also complained they had sore throat and pain in the abdomen and 14.58 % of omeprazole users had pain in the stomach. Besides, chest tightness (52.08 %) and shortness of breath (14.58 %) like cardiac side effects were only common to the esomeprazole users. While considering the genders comparison, for all generic administration of PPIs, side effects such as headache, abdominal discomfort and pain in the abdomen were most common in the male subjects whereas, insomnia, dizziness and nausea were common among the female respondents (Table 8). Outcomes for all generic administration of PPI_s_ are statistically significant (p < 0.05) during their subgroup comparison except for ranitidine and omeprazole groups.

**Table 8:** Some common side effects of antiulcerant therapies (PPIs and H2-Blocker).

Name of the antiulcerant	Common side effects	Number of cases (Percentage)	Case per gender (male/female) (Percentage)	P-value (between genders)	SEM (between subgroups)	P-value (between subgroups)
**H2-Blocker**						
**Ranitidine(Short- and long-term usage)**	Constipation	5 (50.00)	3/2 (66.67/33.33)	0.205 ^NS^	11.58	0.022^NS^
	Flatulence	1 (10.00)	1/0 (100/0.00)	0.500 ^NS^		
	Abdominal discomfort	8 (80.00)	6/2 (75.00/25.00)	0.295 ^NS^		
	Insomnia	4 (40.00)	3/1 (50.00/50.00)	–		
	Dizziness	3 (30.00)	3/0 (100.00/0.00)	0.500 ^NS^		
**Proton pump inhibitors**						
**Omeprazole(Short- and long-term usage)**	Headache	78 (60.94)	57/21 (73.08/26.92)	0.275 ^NS^	25.78	0.403^NS^
	Nausea	12 (9.38)	7/5 (58.33/41.67)	0.105 ^NS^		
**Esomeprazole(Short- and long-term usage)**	Headache	6 (12.50)	5/1 (83.33/16.67)	0.374 ^NS^	6.31	0.019*
	Flatulence	6 (12.50)	2/4 (33.33/66.67)	0.205 ^NS^		
	Stomach pain	7 (14.58)	4/3 (57.14/42.85)	0.090 ^NS^		
	Shortness of breath	7 (14.58)	5/2 (71.43/28.57)	0.258 ^NS^		
	Chest tightness	25 (52.08)	19/6 (76.00/24.00)	0.305 ^NS^		
	Nausea	11 (22.92)	6/5 (54.54/45.46)	0.058 ^NS^		
**Rabeprazole(Short- and long-term user)**	Headache	8 (100.00)	7/1 (87.50/12.50)	0.410 ^NS^	8.84	0.003**
	Nausea	6 (75.00)	3/3 (50.00/50.00)	–		
	Pain in the abdomen	5 (62.50)	4/1 (80.00/20.00)	0.344 ^NS^		
	Sore throat	5 (62.50)	3/2 (60.00/40.00)	0.126 ^NS^		
**Pantoprazole(Short- and long-term user)**	Headache	6 (100.00)	4/2 (66.67/33.33)	0.205 ^NS^	17.88	0.034*
	Nausea	5 (83.33)	3/2 (60.00/40.00)	0.126 ^NS^		
	Dizziness	3 (50.00)	3/0 (100/0.00)	0.500 ^NS^		

Values are represented by a percentage and SEM calculated for the groups; the total number of respondents was 190, *p < 0.05, **p < 0.01; short-term usage (> 3 to < 5 years), and long-term usage (> 5 years). NS stands for statistically not significant at a p-value > 0.05.

### 3.6 Musculoskeletal side effects and bone pain in proton pump inhibitor (PPI) users

During this survey, ranitidine (only H2-blocker recorded) users were not reported with any musculoskeletal side effects. Table 9 represents the data on musculoskeletal side effects for all generic administration of PPIs. Between the generic administration, all the PPI users have tremors and muscle weakness like musculoskeletal side effects. Meanwhile, the omeprazole and rabeprazole users were also suffering from some other musculoskeletal side effects in the following order: spasms of the hands and feet > muscle aches > flank pain (Table 9). However, only omeprazole users had numbness (22.31 %) like musculoskeletal side effects. All the findings are statistically significant (p < 0.05) in their subgroup comparison whereas male respondents take lead over the female respondents (Table 9).

**Table 9:** Musculoskeletal side effects in proton pump inhibitors (PPIs) medications.

Drug name	Musculoskeletal side effects	Number of cases (Percentage)	Case per gender (male/female) (Percentage)	P-value (between genders)	SEM (between subgroups)	P-value (between subgroups)
**Omeprazole(Short- and long-term user)**	Muscle Weakness	90 (70.31)	61/29 (67.78/32.22)	0.218 ^NS^	9.66	0.010**
	Spasms of hands and feet	47 (36.72)	30/17 (63.83/36.17)	0.172 ^NS^		
	Muscle aches	38 (29.69)	25/13 (65.79/34.21)	0.195 ^NS^		
	Flank pain	27 (21.09)	16/11 (59.26/40.74)	0.117 ^NS^		
	Numbness	26 (20.31)	20/6 (76.92/23.08)	0.314 ^NS^		
	Tremor	22 (17.19)	16/6 (72.73/27.27)	0.271^NS^		
**Esomeprazole(Long-term user)**	Muscle weakness	23 (47.91)	17/6 (73.91/26.09)	0.284 ^NS^	7.21	0.039*
	Spasms of hands and feet	17 (35.42)	14/3 (82.35/17.65)	0.366 ^NS^		
	Tremor	11 (22.92)	9/2 (81.82/18.18)	0.361 ^NS^		
**Rabeprazole(Short- and long-term user)**	Tremor	8 (100.00)	6/2 (75.00/25.00)	0.295 ^NS^	7.91	0.000***
	Muscle weakness	8 (100.00)	5/3 (62.50/37.50)	0.156 ^NS^		
	Muscle aches	8 (100.00)	5/3 (62.50/37.50)	0.156 ^NS^		
	Flank pain	6 (75.00)	5/1 (83.33/16.67)	0.374 ^NS^		
	Spasms of hand and feet	5 (62.50)	4/1 (80.00/20.00)	0.344 ^NS^		
**Pantoprazole(Short- and long-term user)**	Tremor	6 (100.00)	4/2 (66.67/33.33)	0.205 ^NS^	16.67	0.038*
	Muscle aches	6 (100.00)	4/2 (66.67/33.33)	0.205 ^NS^		
	Muscle weakness	3 (50.00)	2/1 (66.67/33.33)	0.205 ^NS^		

Values are represented by a percentage and SEM calculated for the groups; the total number of respondents was 190, *p < 0.05, **p < 0.01, ***P < 0.005, NS stands for statistically not significant at p value> 0.05; antiulcerant users: short term usage (> 3 to < 5 years), long term usage (> 5 years).

Besides these musculoskeletal side effects, PPI users also had some localized bone pains. Table 10 represents the types of pains that PPI users had during the treatment period. During this survey, respondents replied they had pain in three different sides: low back pain, neck pain and knee joint pain. Between the generic administration of PPIs, generation of pain in all three sides has been found for omeprazole, pantoprazole and rabeprazole users, whereas pantoprazole users have pains in all three sites with the highest prevalence compared to other generic administration of PPIs. Meanwhile, rabeprazole users became the 2nd most prevalent group for the neck (25 %) and knee joint pains (25 %). Though neck pain was less common in the esomeprazole users whereas 2nd most prevalent group for low back pain (68.75 %). Besides, the subgroup comparison of outcomes from rabeprazole and pantoprazole users is statistically significant (P < 0.05).

**Table 10:** Incidences of pain in the proton pump inhibitors (PPIs) users in the different bone sites.

Drug name	Types of pain	Number of cases (Percentage)	Case per gender (male/female) (Percentage)	P-value (between genders)	SEM (between subgroups)	P-value (between subgroups)
**Omeprazole(Short- and long-term user)**	Low back pain	74 (57.81)	64/10 (86.49/13.51)	0.401 ^NS^	17.02	0.295^NS^
	Neck pain	13 (10.16)	10/3 (76.92/23.08)	0.314 ^NS^		
	Knee joint pain	5 (3.91)	3/2 (60.00/40.00)	0.126 ^NS^		
**Esomeprazole(Long-term user)**	Low back pain	33 (68.75)	25/8 (75.76/24.24)	0.303 ^NS^	32.29	0.461^NS^
	Neck pain	2 (4.17)	2/0 (100.00/0.00)	0.500 ^NS^		
**Rabeprazole(Short- and long-term user)**	Low back pain	3 (37.50)	3/0 (100.00/0.00)	0.500 ^NS^	4.17	0.020*
	Neck pain	2 (25.00)	2/0 (100.00/0.00)	0.500 ^NS^		
	Knee joint pain	2 (25.00)	1/1 (50.00/50.00)	–		
**Pantoprazole(Short- and long-term user)**	Low back pain	6 (100.00)	4/2 (66.67/33.33)	0.205 ^NS^	11.11	0.020*
	Neck pain	4 (66.67)	4/0 (100.00/0.00)	0.500 ^NS^		
	Knee joint pain	4 (66.67)	2/2 (50.00/50.00)	–		

Values are represented by a percentage and SEM is calculated for the groups. *p < 0.05 and NS stands for statistically not significant at p value > 0.05; the table is based on the positive outcomes of the volunteers; respondents with negative and unknown responses constitute the rest percentage within the vicinity of the present data sheet; the total numbers of respondents was 190.

### 3.7 The response might have a link to the musculoskeletal dysfunction

Table 11 describes the respondent’s response to some queries that might have some link to the musculoskeletal dysfunctions. The results showed that the majority (57 %) of individuals reporting to be active “all of their life” 33 % of respondents said they were active “when they were younger” whereas 8 % of them said “no”. Meanwhile, the majority (90 %) of respondents have found without any falling incidences whereas 10 % of antiulcerants users replied they have fallen experience within the last 5 years (all the respondents belonged to PPIs receiving group). Finally, 23 % of antiulcerants users have an unsteady feeling when getting out of a chair or walking. The study data hasn’t come with any significant difference between the subgroup comparison.

**Table 11:** The response might have a link to musculoskeletal dysfunction.

Questions	Subgroup	Percentage	Case per gender (male/female) (Percentage)	P-value (between genders)	SEM (between subgroups)	P-value (between subgroups)
**Physically active individual?**	No	20 (10.00)	15/5 (75.00/25.00)	0.295^NS^	13.57	0.133 ^NS^
	Yes, all my life	114 (57.00)	64/50 (56.14/43.86)	0.078^NS^		
	Yes, when I was younger	66 (33.00)	45/21 (68.18/31.82)	0.222^NS^		
**Fallen in the past years?(without any fracture incidences)**	Yes	20 (10.00)	16/4 (80.00/20.00)	0.344^NS^	28.48	0.362 ^NS^
	No	180 (90.00)	108/72 (60.00/40.00)	0.126^NS^		
	Don’t know	0 (0.00)	0 (0.00)	–		
**Feeling unsteady (When get out of a chair or walk)?**	Yes	46 (23.00)	32/14 (69.57/30.43)	0.238^NS^	22.82	0.282 ^NS^
	No	156 (77.00)	94/62 (60.26/39.74)	0.129^NS^		
	Don’t know	0 (0.00)	0 (0.00)	–		

Values are represented by a percentage and SEM calculated for the groups; the total number of respondents was 200; NS stands for statistically not significant at a p-value > 0.05.

### 3.8 Clinical history of skeletal and other pathological conditions in respondents taking antiulcerants

Besides, the GI disorders, respondents also had some skeletal and other problems within the period of taking anti-ulcerants for GI problems. About 15 % of the respondents were suffering from osteopenia while only six respondents were diagnosed with serious problems in bone mineral density (osteoporosis) (within the last three years of taking PPIs treatment) (Table 12). Meanwhile, 21 %, 18.5 %, and 16 % of respondents claimed they were suffering from hypocalcemia, hypomagnesemia and vitamin-B12 deficiency where hypocalcemia takes place over hypomagnesemia and V-B12 deficiency (Table 7). While considering the disorders other than GI and skeletal problems patients also suffer from disorders such as; hypertension (33 %), diabetics (26 %), rheumatoid arthritis (10.5 %), anemia (6 %) and renal dysfunctions (5 %) (Table 12).

**Table 12:** Clinical history of skeletal and other pathological conditions in respondents taking antiulcerants.

Case	Subgroup	Number of cases (Percentage)	Case per gender (male/female) (Percentage)	P-value (between genders)	SEM (between subgroups)	P-value (between subgroups)
**Problems related to the skeletal system**	Osteopenia	15 (7.50)	9/6 (60.00/40.00)	0.194 ^NS^	5.01	0.258^NS^
	Osteoporosis	6 (3.00)	4/2 (60.67/33.33)	0.215 ^NS^		
**Vitamin and mineral deficiencies**	Hypocalcemia	42 (21.00)	25/17 (59.52/40.48)	0.120 ^NS^	1.44	0.006**
	Hypomagnesemia	37 (18.50)	22/15 (59.46/40.54)	0.119 ^NS^		
	Vitamin B12 deficiency	32 (16.00)	22/10 (68.75/31.25)	0.228 ^NS^		
**Other types**	Hypertension	66 (33.00)	45/21 (68.18/31.82)	0.221 ^NS^	5.66	0.047*
	Diabetes	52 (26.00)	38/14 (73.08/26.92)	0.243 ^NS^		
	Rheumatoid arthritis	21 (10.50)	16/5 (76.19/23.81)	0.307 ^NS^		
	Anemia	12 (6.00)	2/10 (16.67/83.33)	0.374 ^NS^		
	Renal Disease	10 (5.00)	9/1 (90.00/10.00)	0.335 ^NS^		

Values are represented by a percentage and SEM calculated for the groups; the total number of respondents was 200. The table is based on the positive outcomes of the volunteers. Respondents with negative and unknown responses constitute the rest percentage within the vicinity of the present datasheet, only one respondent from H2-blockers users has hypomagnesemia and hypocalcemia conditions whereas the rest of the respondents belong to the PPIs recipient group; *p < 0.05, **p < 0.01; NS stands for statistically not significant at p-value > 0.05.

### 3.9 Respondent's family history of osteoporosis and fracture

Table 13 describes the respondent’s family (paternal and maternal) history of fractures. Among the participants, 12 % and 7 % of respondents replied they have a history of maternal and paternal fractures respectively when they were more than 40 years old. While focusing on the maternal fracture’s history, the data shows that 41.67 % of fractures had happened in the wrist (maternal case) while the fracture incidences to other bone sites had taken the following order elbow > femur > foot > hand (Table 13).

**Table 13:** Respondent's family history of osteoporosis and fractures.

Case	Types of fractures	Number of cases (percentage)	Case per gender (male/female) (percentage)	P-value (between genders)	SEM (between subgroups)	P-value (between subgroups)
**Maternal (n = 24)**	Wrist	10 (41.67)	8/3 (80.00/20.00)	0.344 ^NS^	5.81	0.026*
	Elbow	5 (20.83)	4/1 (80.00/20.00)	0.344 ^NS^		
	Femur	4 (16.67)	4/0 (100.00/0.00)	0.500 ^NS^		
	Foot	3 (12.5)	2/1 (66.67/33.33)	0.205 ^NS^		
	Hand	2 (8.33)	2/0 (100.00/0.00)	0.500 ^NS^		
**Paternal(n = 14)**	Femur	9 (64.29)	8/1 (88.89/11.11)	0.422 ^NS^	15.62	0.166 ^NS^
	Shoulder	3 (21.43)	2/1 (66.67/33.33)	0.205 ^NS^		
	Hip	2 (14.28)	2/0 (100.00/0.00)	0.500 ^NS^		

Values are represented by a percentage and SEM calculated for the groups; the table only represents the positive outcomes from the volunteers. * for p < 0.05; NS stands for statistically not significant at p-value > 0.05.

Furthermore, while comparing the responses about the paternal history of fracture risk, the highest number of respondents said about femur fractures while the history of shoulder and hip fractures have taken 21 % and 15 % scores respectively.

### 3.10 Other medications status

Table 14 describes the other medication status of respondents. The Table 9 shows that 14 % of respondents have a history of taking the antihypertensive drug. About 7 % of respondents have taken oral hypoglycemic drugs for diabetic ailments while 6 % of respondents were taking non-steroidal anti-inflammatory drugs (NSAIDs) for their pathological pains. Finally, very few cases have been found who were taking the antidepressant drug and calcium supplements.

**Table 14:** Other medications history.

Class of the drug	Number of cases (percentage)	Case per gender (male/female) (percentage)	P-value (between the gender)	SEM (between the groups)	P-value (between the groups)
**Antihypertensive**	28 (14.00)	22/6 (78.57/21.43)	0.330^NS^	2.06	0.034*
**Oral hypoglycemic drugs**	14 (7.00)	10/4 (71.43/28.57)	0.258^NS^		
**Non-steroidal anti-inflammatory drugs (NSAID)**	12 (6.00)	8/4 (66.67/33.33)	0.205^NS^		
**Antidepressant drugs**	6 (3.00)	5/1 (83.33/16.67)	0.374^NS^		
**Calcium supplement**	5 (2.50)	2/3 (40.00/60.00)	0.126^NS^		

Values are represented by a percentage and SEM calculated for the groups; the table only represents the positive outcomes from the volunteers; the total number of respondents was 200; NS stands for statistically not significant at p-value > 0.05.

## 4 Discussion

This prospective study examined the relationships between acid-suppressive medication use and the incidence of musculoskeletal side effects among 200 respondents from five different hospitals in Bangladesh. In a country like Bangladesh, people are still suffering from mineral and vitamin deficiencies [21]. Many studies reported the linkage between minerals and vitamin deficiencies and musculoskeletal problems [22], [23], [24]. Thus, long-term administration of any medications with a connexion of mineral and vitamin deficiencies in a country like Bangladesh might get the situation worst and hamper the quality of an individual's life while also increasing the hardships of individuals already having acute to chronic musculoskeletal problems.

During this survey, respondents have chosen between PPIs and H2-blockers users for several GI problems where most of them were PPIs users (95 %). This large usage difference between these two antiulcerants might be due to the good treatment success with greater inhibition of gastric acid secretion properties of PPIs at low doses while safety concerns for the H2-blocker use in some cases [6], [7], [8] and the recent report of the presence of unacceptable level of N-Nitrosodimethylamine, hepatotoxic and carcinogenic agents, in H2-Blockers, might also decrease the number of H2-blockers prescriptions nowadays [9], [10].

The study computed several influencing factors such as age, gender, BMI, dietary intake, cigarette smoking and alcohol status and a few other parameters, but we didn’t observe any significant differences between these parameters that can affect the study outcomes (Table 1 and Table 2). After computing all potential confounding factors, long-term use of PPIs in study subjects was found associated with multiple musculoskeletal problems while few cases of site-specific bone pains, osteopenic and osteoporotic incidences have been recorded (Table 9, Table 10 and Table 12). Meanwhile, minerals (calcium and magnesium) and vitamin B12 deficiencies in many cases increase the importance of this current study (Table 12).

Both studied antiulcerants have some previous track of musculoskeletal systems disturbance [25], [26], [27], [28], [29]. During the current study, only PPI users have identified some musculoskeletal side effects and minerals and vitamin B_12_ deficiencies (Table 9 and Table 12). The musculoskeletal dysfunctions primarily appear with symptoms like fatigue, stiffness, numbness, muscle weakness, tremor, unsteadiness and pains on different bone sites. Meanwhile, if the symptoms are ignored and remained untreated then serious skeletal problems such as osteomalacia, osteonecrosis, osteopenia, and osteoporosis might occur afterwards [30], [31], [32], [33], [34], [35].

Numerous epidemiological and cohort studies have provided evidence of an association between long-term PPI use and increased musculoskeletal abnormalities among users [36], while several studies on the animal model also replicate PPI's possible roles in osteoporosis and fracture risk [37], [38]. Overall, proton pump inhibitors (PPIs) covalently bind with hydrogen-potassium stimulated adenosine triphosphate (H^+^ K^+^-ATPase) ion exchanger and lead to a profound inhibition of gastric acid secretion, thus, impairing the number of minerals and vitamins absorption while also can dysregulate the hormonal functions [39]. Several previous animal and human studies have shown that alteration of gastric acid secretion with the PPIs medications can affect the absorption of minerals and vitamins [40], [41], [42]. Thus, interfering with the bone metabolic and remodeling functions of osteoblast and osteoclast cells [40], [41], [42]. Our current data showed that a significant number of PPI users had musculoskeletal side effects and site-specific bone pains (Table 9 and Table 10).

**Fig. 1:**
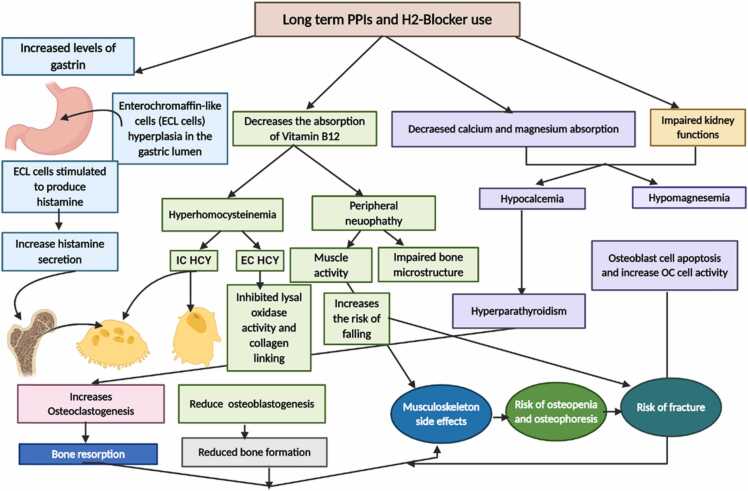
Possible pathways for antiulcerants (PPIs and H2-Blocker) inducing musculoskeletal side effects and increased fracture risks (Abbreviations: IC HCY–intracellular homocysteine; EC HCY–extracellular homocysteine; Ca–calcium; Mg–magnesium; BMD–bone mineral density; OB–osteoblast; OC–osteoclast).

The possible mechanism is PPIs administration can reduce the epithelial acidification and thereby increase the colonial pH and fate to the reduction of Mg^2+^, Ca^2+^ and other minerals absorption [43]. The PPIs induced hypocalcemia has received attention recently and many human and animal-based studies have been conducted so far to test this hypothesis [25], [26], [27], [28], [29]. Overall, the results showed long term with PPI treatment outbreaks the risk of hypocalcemia-induced skeletal dysfunctions [25], [26], [27], [28], [29]. Previous studies demonstrated that long-term PPIs lead to calcium malabsorption and may trigger secondary hyperparathyroidism in response [44], [45], [46]. Excessive PTH release in secondary hyperparathyroidism condition in turn upshot the calcium concentration in blood and manifested with symptoms such as muscle aching and weakness (Fig. 1) [47], later on, severe skeletal damage such as bone softening (osteomalacia), osteopenia, osteoporosis and fractures in different bone sites might happen [48].

Nowadays, proton-pump inhibitor-induced hypomagnesemia is one of the most recognized side effects of PPIs and the situation becomes worst when these drugs are given to the patients for a longer duration with frequent dosing. A list of previous cases demonstrated that long-term PPIs medications were linked to hypomagnesemia whereas the situation was resolved by stopping PPIs therapy and repeated by reintroducing PPI therapy [30], [31], [32], [33], [34], [35]. Meanwhile, hypomagnesemia in certain cases was found associated with hypokalemia and/or hypercalcemia like mineral deficiencies [33], [35].

In many previous studies, proton pump inhibitors (PPIs) have proven their negative role in vitamin B-12 absorption [49], [50], [51], [52]. However, they can only impair the absorption of protein-bound VB_12_ but have no action on crystalline VB_12_ absorption [49], [50], [51], [52]. Many previous *in-vitro* and *in-vivo* studies validate the importance of normal regulation and function of vitamin B-_12_ in bone health [53], [54], [55], [56]. A previous *in-vitro* study showed that vitamin B-12 is important for the expression and function of alkaline phosphatase (ALP) in osteoblast proliferation and bone mineralization [56]. Meanwhile, a previous human study by Carmel et al. showed that vitamin B-12–deficient patients may experience bone mineral content (BMC) and bone mineral density (BMD) suppression as the expression of alkaline phosphatase (ALP) and osteocalcin (OCN) is decreased with the vitamin B-12 deficiency [55] whereas these situations are improved and recovered with vitamin B_12_ supplementation [55]. Similar findings were also revealed by many previous studies on human subjects [53], [54]. Thus, long-term PPIs usage associated with vitamin-B12 deficiency might alter the osteoblast proliferation and mineralization and thus increase the incidence of skeletal abnormalities and fracture risks.

Furthermore, several previous publications revealed that individuals with a familiar history of bone fractures are considered the risk group for experiencing fractures in future [57], [58], [59], [60], thus clinical suggestions, in that case, are any agents and drugs with a risk history of pathological bone loss should be carefully administered with revised dose and doses frequency, if there is any alternatives treatment than should be avoided [57], [58], [59], [60]. Our present survey data also recorded a few cases of those who have a family history of fractures (Table 13), therefore, care should be taken while prescribing PPIs to individuals with a family history of fractures.

In summary, the current study data suggest that long-term PPI treatment has some linkage to musculoskeletal problems. With the current findings, it is evident that anti-ulcerant therapies might develop skeletal problems in long-term usage while may also delaying the healing of osteoporosis or fractures in patients. Thus, awareness and precautions should be raised among the patients and clinicians for the careful administration of PPIs to individuals, especially to patients already suffering from bone disorders and family history of fractures. It is difficult to find a treatment/medication that would bypass the musculoskeletal side effects of PPIs, as there are no such guidelines in the literature. However, patients should avoid long-term or unprescribed use of PPIs, and should monitor their bone health if they need to take it for longer than usual. If a patient needs additional medication or supplements to maintain their musculoskeletal health, they should contact a doctor. However, the dietary uptake rich in essential minerals, phosphates and vitamins could be helpful in this aspect.

## 5 Limitations

The present data only covered 200 respondents from five different hospitals in Bangladesh. So, the current data seems not enough to give the whole scenario of musculoskeletal problems of all antiulcerants users in Bangladesh. So, a cohort study in a large population size with a more descriptive questionnaire should be conducted to have a complete scenario of the risk between the antiulcerants users and probable complications to musculoskeletal systems.

## 6 Conclusion

PPIs are relatively safe and less toxic in comparison to other antiulcerants but the risk of osteoporosis and fracture in long-term administration should not be overlooked. Thus, suggestions from the current study are consciousness should be created among the participants and clinicians in the hospital so that the appropriate prescription of PPIs will improve patient satisfaction while the incidence of osteoporosis and fractures can be avoided. The current study represents the outcomes from a small group population hence a multi-centered investigation and clinical studies are recommended to obtain more conclusive and valid results that would better reflect the usage and health-hazardous patterns of anti-ulcerant therapy in Bangladesh.

## CRediT authorship contribution statement

All authors meticulously reviewed the draft and gave their consent to publish this article.

## Ethical consideration

Consent was taken from all the study participants before the interview. Information taken from the participants was preserved confidentially.

## Declaration of Competing Interest

The authors declare that they have no known competing financial interests or personal relationships that could have appeared to influence the work reported in this paper.

## Data Availability

Data will be made available on request.
